# Supplementary material for: Causal Relationship between Aging and Anorexia Nervosa: A White-Matter-Microstructure-Mediated Mendelian Randomization Analysis
Source: Biomedicines. 2024 Aug 16;12(8):1874. doi: 10.3390/biomedicines12081874 (PMC11351342; doi:10.3390/biomedicines12081874)
Supplement: Supplementary file 1 [file biomedicines-12-01874-s001.zip › Supplementary Figure/Figure S7/Single SNP plot of MR analysis for ubm-b-1969 to AN.pdf]

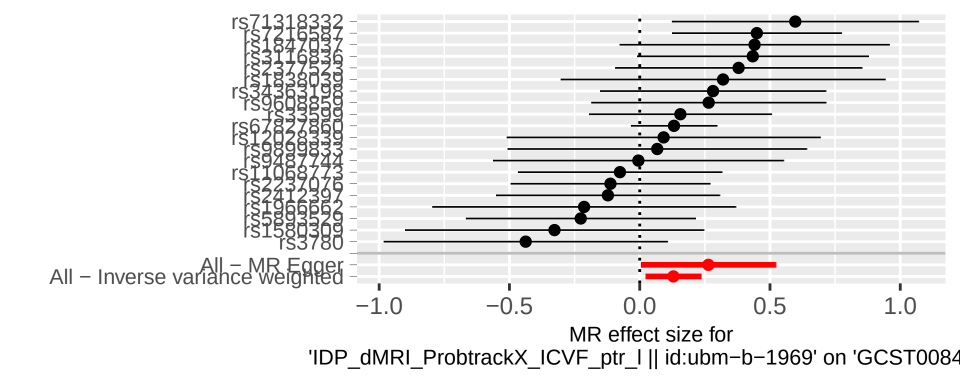
rs71318332
rs7216587
rs1847037
rs3116836
rs2377523
rs1838039
rs34363198
rs9608859
rs33599
rs67827860
rs12028339
rs9899833
rs9487744
rs11068773
rs2237076
rs2412397
rs1966662
rs5893529
rs1580309
rs3780
All − MR Egger
All − Inverse variance weighted
−1.0
−0.5
0.0
0.5
1.0
MR effect size for
'IDP_dMRI_ProbtrackX_ICVF_ptr_l || id:ubm−b−1969' on 'GCST0084
